# Assessing serum anti-nuclear antibodies HEp-2 patterns in synucleinopathies

**DOI:** 10.1186/s12979-024-00453-0

**Published:** 2024-07-18

**Authors:** Jonas Folke, Marie Skougaard, Trine-Line Korsholm, Anne-Line Strange Laursen, Lisette Salvesen, Anne-Mette Hejl, Sara Bech, Annemette Løkkegaard, Tomasz Brudek, Sisse Bolm Ditlev, Susana Aznar

**Affiliations:** 1 Centre for Neuroscience & Stereology, Department of Neurology, Bispebjerg and Frederiksberg Hospital, Copenhagen University Hospital, Copenhagen, Denmark; 2 Copenhagen Center for Translational Research, Bispebjerg and Frederiksberg Hospital, Copenhagen University Hospital, Copenhagen, Denmark; 3 Department of Clinical Immunology, Aarhus University Hospital, Aarhus, Denmark; 4 Department of Neurology, Bispebjerg and Frederiksberg Hospital, Copenhagen University Hospital, Copenhagen, Denmark; 5 Department of Clinical Medicine, Faculty of Health and Medical Sciences, University of Copenhagen, Blegdamsvej 3B, Copenhagen Ø, DK-2100 Denmark

**Keywords:** Autoantibodies, Synucleinopathies, Movement disorders, Antinuclear antibodies

## Abstract

This study investigates the presence of antinuclear antibodies (ANA) in three primary synucleinopathies – Parkinson’s disease (PD), multiple system atrophy (MSA), and dementia with Lewy bodies (DLB), compared to healthy controls. Autoinflammatory disorders typically involve the immune system mistakenly attacking the body’s own cells and start producing ANA. There is an increasing body of evidence that immune-mediated inflammation is a pathological feature linked to synucleinopathies. To investigate whether this could be autoimmune mediated we analyzed for ANA in the plasma of 25 MSA, 25 PD, and 17 DLB patients, along with 25 healthy controls, using the ANA HEp-2 indirect immunofluorescence antibody assay (ANA HEp-2 IFA). Contrary to initial expectations, results showed ANA HEp-2 positivity in 12% of PD, 8% of MSA patients, 18% of DLB patients, and 17% of healthy controls, indicating no increased prevalence of ANA in synucleinopathies compared to age-matched healthy individuals. Various ANA HEp-2 patterns were identified, but no specific pattern was associated with individual synucleinopathies. We conclude hereby that synucleinopathies are not associated with detectable presence of ANA in plasma.

**Supplementary Information:**

The online version contains supplementary material available at 10.1186/s12979-024-00453-0.

## Introduction

Synucleinopathies represent a clinically and pathological diverse group of aging-related neurodegenerative disorders, unified by the accumulation of insoluble α-synuclein inclusions and cell death in the brain. Broadly categorized into Lewy body diseases (LBD) and Multiple System Atrophy (MSA), these disorders differ primarily in the cellular distribution of aggregated α-synuclein, neuronal in LBD and oligodendrocytic in MSA. Parkinson’s disease (PD), the most prevalent LBD, is primarily a motor disorder, marked by symptoms like bradykinesia, rigidity, rest tremor, and postural instability, though non-motor symptoms also can be present. Dementia with Lewy bodies (DLB), the second LBD, predominantly manifests as dementia, cognitive dysfunction, and visual hallucinations, often accompanied by parkinsonism, but not necessarily by prominent motor dysfunction. MSA, in contrast, is a movement disorder characterized by autonomic failure, levodopa-unresponsive parkinsonism, cerebellar ataxia, pyramidal signs, and various non-motor symptoms (reviewed in [1]).

The etiology behind synucleinopathies and the cause of neuronal death remains elusive. Over the past decade, various hypotheses have been proposed, ranging from dysregulation in α-synuclein processing and cellular quality control mechanisms to mitochondria dysfunction and oxidative stress due to imbalances in reactive oxygen species production and metabolism [2, 3]. Recently, the role of immune response dysregulation and neuroinflammation in these diseases has garnered attention [4–6]. Initial research focused on microglia, the brain’s immune cells [7–9]. However, newer studies indicate a significant involvement of the peripheral immune system in disease pathophysiology [10–12]. The idea of autoimmune mechanisms in synucleinopathies was first proposed by the finding of antibody cross-reactivity recognizing Epstein-Barr virus and α-synuclein in PD brains [13], hinting at molecular mimicry leading to α-synuclein oligomerization in genetically susceptible individuals [14]. A later finding by Sulzer et al [15] showing that α-synuclein epitopes can trigger T-cells responses, primarily CD4 helper responses but also CD8 cytotoxic responses, in PD patients bolstered the view of PD as a disorder with a potential autoimmune component. More recently, it has been shown that T-cell brain infiltration precedes α-synuclein aggregation, with tissue resident memory CD8 T-cells responding to unidentified epitopes [16].

Further supporting this, the presence of activated CD4 T-cells in the brains of DLB patients have been reported correlating with neuroaxonal damage [17]. MSA has been linked genetically to autoimmune disorders like inflammatory bowel disease [18] and primary Sjögren’s syndrome [19]. Comparative studies of cerebrospinal fluid in DLB, MSA and PD patients reveal stronger activation of immune pathways in DLB and MSA than in PD, which aligns well with DLB and MSA presenting a much more severe disease trajectory than PD [20].

Anti-nuclear antibodies (ANA) are a diverse group of autoantibodies targeting cellular (nuclear and cytoplasmic) antigens. They are commonly used in clinical practice to diagnose autoimmune diseases, whose causes are often unknown [21]. These diseases include Systemic lupus erythematosus (SLE) and Sjögren’s syndrome [22]. The prevalence of ANA positivity among healthy individuals can vary widely, ranging from 7 to 25%, depeding on factors such as the demographic characteristics and the specific screening methods [23]. ANA-positivity in asymptomatic, seemingly healthy individuals has been reported to be associated with latent inflammatory conditions and immune dysfunctions, which can include changes in T-cell populations [21, 24]. In addition to their role in systemic autoimmune conditions, ANA have been detected in central nervous system (CNS) related autoimmune inflammatory disorders, such as neuromyelitis optica [25]. Here, increased ANA levels are associated with increased disease activity and more severe disability [25]. In the context of neurodegenerative disorders like Alzheimer’s disease and PD, elevated levels of anti-double stranded DNA immunoglobulins (IgGs) have been reported [26, 27]. However, the presence of ANA in MSA and DLB has not yet been investigated.

The aim of our study was to investigate whether ANA are present in blood samples from a cohort comprising 25 PD patients, 25 MSA patients, 17 DLB patients, and 25 healthy controls employing an indirect immunofluorescence assay using HEp-2 cells (HEp-2 IFA).

## Materials and methods

### Demographics

Plasma samples from PD (*N* = 25), MSA (*N* = 25), DLB (*N* = 17), and healthy controls (*N* = 25) were retrieved from Centre for Neuroscience and Stereology Research Biobank, Bispebjerg-Frederiksberg Hospital (Table 1). Patients met the diagnostic criteria with a certainty level of probable or higher [28–30]. Each participant provided written informed consent for the experiment and sampling for biobanking, adhering to the World Medical Association Declaration of Helsinki. The study was approved by the regional ethical committee of the Capital Region of Denmark (H-15,016,232) and the data protection agency (P-937-2020).

### ANA HEp2 IFA

The commercially available NOVA Lite^®^ HEp-2 ANA Kit with DAPI (Inova Diagnostics; lot nr. #084429), which includes human epithelial cells (HEp-2) was applied to evaluate the presence of ANA in the plasma of included patients and controls. Automated preparation of the ANA HEp-2 slides with patient sample was conducted on the QUANTA-Lyser 160 (Werfen, ES) according to manufacturer’s instructions. A dilution of 1:160 was utilized, established by the performing laboratory as corresponding to the 95th percentile among healthy controls, in accordance with international guidelines [23]. Briefly, preparation comprised incubation of fixed HEp-2 cells with diluted patient sample. After incubation, the HEp-2 cells were washed and the polyclonal anti-human IgG/FITC was used as secondary detection antibody. Automated imaging of ANA HEp-2 slides was performed by the NOVA View 2.0 IFA Microscope (Werfen, ES) and all images retrieved from microscopy were transferred for analysis in QUANTA Link (Werfen, ES). ANA HEp-2 IFA patterns were assigned and validated in accordance with International Consensus on ANA Patterns (ICAP) [31] by medical doctors with more than 10 years of experience in reading ANA HEp-2, who were unaware of diagnosis at the time of reading.

### Statistics

Demographics were analyzed by one-way ANOVA; Kruskall-Wallis; Welch ANOVA; chi-squared; Mann-Whitney test. ANA HEp-2 patterns were compared across groups by Fisher’s exact test. P-values below 0.05 were considered significant.

## Results

### Synucleinopathies were not associated with specific ANA

**Fig. 1:**
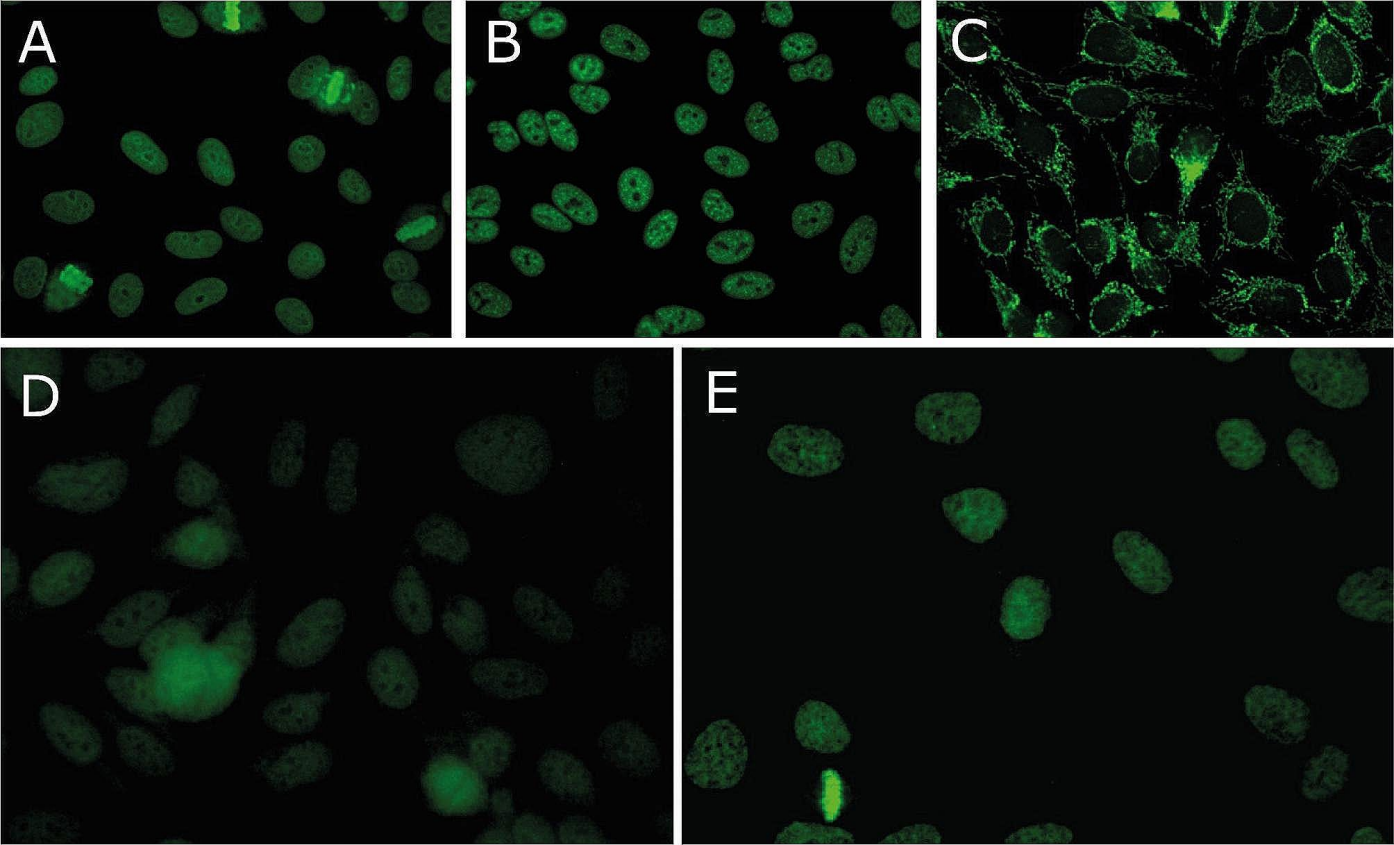
ANA HEp-2 IFA patterns. An ANA HEp-2 indirect immunofluorescence antibody assay (IFA) was applied to screen for autoantibodies in patients with Parkinson’s disease, multiple system atrophy, dementia with Lewy bodies, and healthy controls. ANA HEp-2 IFA patterns were assigned in accordance with International Consensus on ANA patterns (ICAP) and included (**A**) nuclear homogeneous (AC-1), (**B**) nuclear large/coarse speckled (AC-5), (**C**) Cytoplasmic reticular/mitochondria-like (AC-21), (**D**) weak nuclear fine speckled (AC-4), and (**E**) weak nuclear dense fine speckled (AC-2)

ANA HEp-2 IFA (Fig. 1) was used to assess for ANA in patients diagnosed with synucleinopathies, including PD patients (*N* = 25), MSA patients (*N* = 25), DLB patients (*N* = 17), and healthy controls (*N* = 25). ANA HEp-2 was positive in 8/67 (12%) patients with synucleinopathies and 4/17 (24%) healthy controls. Of the PD patients, 3/25 (12%) were ANA HEp-2 positive, exhibiting ANA patterns of *nuclear homogenous* (8%) and *cytoplasmic*,* reticular/mitochondria-like* (4%). For MSA patients, 2/25 (8%) were ANA HEp-2 positive, with ANA patterns of 8% *nuclear homogenous* and 4% *nuclear large/coarse speckled*. Meanwhile, 3/17 (18%) DLB patients were ANA HEp-2 positive, including ANA patterns of *nuclear dense fine speckled (12%)* and *cytoplasmic*,* reticular/mitochondria-like* (6%) (Table 2). No overall differences in the prevalence of ANA was found between patients with synucleinopathies and healthy controls, Furthermore, healthy controls were overall found to have the same ANA patterns as patients with synucleinopathies. The higher presence of DFS-70 in DLB is not disease related as this ANA is frequently present in healthy individuals [32].

## Discussion

This study explored the presence of ANA in plasma samples from patients suffering from PD, MSA, or DLB to evaluate a potential autoimmune component of synucleinopathies. Autoimmunity has been suggested as a possible pathological mechanism involved in these diseases [14, 17, 26, 33–36]. Therefore, the exploratory method ANA HEp-2 IFA was implemented to determine the presence of ANA-associated nuclear, cytoplasmic, and mitotic autoantibodies. The exploratory approach ensuring higher sensitivity was deemed the most relevant when exploring ANA in non-connective tissue diseases, excluding more specific solid phase assays as no specific autoantibodies have been associated with synucleinopathies.

Several neurological disorders, such as autoimmune encephalitis and myelitis, are associated with autoantibodies targeting neuronal structures [37]. Over the last 20 years, an increasing number of neurological diseases with a previously unsuspected humoral autoimmune component have emerged, with these antibody-associated neurological diseases presenting a wide range of clinical symptoms (37). The previous assumption that the CNS is immune-privileged, protected by the blood-brain barrier, has been increasingly challenged. This shift in perspective is largely due to the realization of a central-peripheral immune interaction influencing the pathogenesis of primary CNS diseases like multiple sclerosis, and neuropsychiatric symptoms in systemic autoimmune conditions such as SLE [38]. The growing recognition of this brain-periphery immune axis has led to exploring its potential pathological involvement in neurological diseases with unknown etiologies and has further spawned the idea that neuron-targeting autoantibodies could serve as biomarkers for neurodegenerative diseases [39]. This presupposition laid the basis for our study.

Previous studies have examined ANA in serum from PD patients, reporting a marginal increase in the presence of anti-phosphatidylserine (PS) and anti-dsDNA IgGs [26]. Here, we did not investigate anti-PS IgGs, and only 8% of PD patients in our cohort were ANA HEp-2 AC-1 positive, the ANA pattern associated with autoantibodies against dsDNA, nucleosomes and histones. However, this can be explained by a smaller cohort in this study. Nevertheless, in our study we included the whole spectrum of ANA (nuclear, cytoplasmic, and mitotic). Additionally, we included not only PD, but also related parkinsonian diseases with a more severe clinical presentation, that, according to our previous studies, have altered IgM and IgG1 levels [40] and increased CNS presence of IgGs [20]. There was no evidence of PD, MSA, or DLB being associated with circulating ANA, and the prevalence of ANA-positive titers was even lower in patients compared to age-matched healthy controls. The prevalence of ANA positive in healthy controls in the study was unexpectedly high which might be explained by the age and gender of the population. It is well-known that the prevalence of ANA positivity increases with age and female gender [41]. Whether lower ANA could indicate a type of “autoimmunodeficiency” is debated [42], and interesting considering that we previously have found a decreased IgG reactivity towards alpha-synuclein in PD and MSA patients [40, 43, 44].

It might be considered a limitation to the study that the examination of ANA were only performed using peripheral blood as discrepancies between peripheral blood and cerebrospinal fluid have been recognized [45]. Still, the absolute autoantibody titers are almost always higher in serum than in CSF [37] Therefore, blood plasma is the obvious first place to look, although, we cannot exclude the presence of ANA in CSF in these patients. Additionally, the study only applied one kit for the ANA HEp-2 analysis and possible variations between ANA HEp-2 kits from different manufacturers should be acknowledged together with the fact that the ANA HEp-2 IFA has variable detection of some autoantibodies and cannot detect all known ANA, such as Sjögren’s syndrome A antibody (SSA). Further, a screening dilution of 1:160 was implemented, which may lead to false negative results in relation to low-titre weak ANA (if in serum as observed in some samples in the study (Fig. 1D and E)). However, considering the disease severity associated with synucleinopathies of included patients, it seems unlikely that low-titre weak ANA could account for the disease, as autoantibody concentrations and thereby fluorescent intensity are associated with disease activity in other autoimmune diseases [25].

Even though synucleinopathies are not associated with ANA, this does not exclude the possibility of an autoimmune component in these disorders, as it is well-known that an absence of autoantibodies does not exclude autoreactive component [46]. Hence, we conclude that the ANA HEp-2 IFA cannot be implemented to support diagnostics of synucleinopathies.

**Table 1:** Demographics

Group	Age*	Sex (M/F)**	Disease duration (years)****	MOCA score***	H&Y****
PD	65.0 (9.0) [47–78]	11/14	8.8 (5.7) [1–26]	26.6 (2.9) [20–30]	2.2 (0.6) [1–4]
MSA	63.5 (8.2) [44–77]	11/14	5.4 (3.5) [0–16]^&^	-	4.4 (0.9) [2–5]^€^
DLB	74.8 (5.7) [62–87]^$;#;¤^	16/1	6.0 (2.4) [2–12]	12.0 (5.8) [6–24] (83%)	2.4 (1.1) [1–5]
NC	67.0 (7.6) [48–81]	11/14	-	-	-
p-values	*p* < 0.001	*p* = 0.003	*p* = 0.043	*P* < 0.001	*p* < 0.001

PD: Parkinson’s Disease. MSA: Multiple System Atrophy. DLB: Dementia with Lewy Bodies. NC: Normal healthy Controls. M: Male. F: Female. H&Y: Hoehn and Yahr scale (7-stage version). Data presented as mean including standard deviation (), and range []. *: One-way ANOVA with Tukey for multiple comparison; **: chi-squared test; ***: Mann-whitney test. ****: Kruskall-Wallis test. $: DLB vs. NC, *p* < 0.05; #: DLB vs. PD, *p* < 0.001; ¤: DLB vs. MSA, *p* < 0.001, €: MSA vs. DLB and PD, *p* < 0.001. &: MSA vs. PD, *p* = 0.037

**Table 2:** ANA HEp-2 patterns detected

Pattern	Pattern code	Antigen association	Parkinson’s Disease (*N* = 25)	Multiple System Atrophy (*N* = 25)	Dementia with Lewy bodies (*N* = 17)	Healthy controls (*N* = 25)	*P*-values*
ANA HEp-2 positive, n (%)			3 (12)	2 (8)	3 (18)	4 (17)	*P* = 0.764
Nuclear homogeneous	AC-1	dsDNA, nucleosomes, histones	2 (8)	1 (4)	0 (0)	2 (8)	*P* = 0.810
Nuclear dense fine speckled	AC-2	DFS-70	0 (0)	0 (0)	2 (12)	0 (0)	*P* = 0.0033
Nuclear fine speckled	AC-4	SSA/Ro, SSB/La, Mi-2, Ku, etc.	0 (0)	0 (0)	0 (0)	1 (4)	*P* > 0.999
Nuclear large/coarse speckled	AC-5	U1RNP, Smith, RNA polymerase III, etc.	0 (0)	1 (4)	0 (0)	1 (4)	*P* > 0.999
Cytoplasmatic reticular (mitochondria-like)	AC-21	PDC-E2/M2, BCOADC-E2, OGDC-E2, E1α subunit of PDC, E3BP/protein X	1 (4)	0 (0)	1 (6)	0 (0)	*P* = 0.552

Only ANA HEp-2 patterns detected in the study and deemed positive were included in table. *: Fisher’s exact test. Percentages in parentheses

### Electronic supplementary material

Below is the link to the electronic supplementary material.

Supplementary Material 1

## Data Availability

No datasets were generated or analysed during the current study.

## Abbreviations

ANA: Antinuclear Antibodies
PD: Parkinson’s disease
MSA: multiple system atrophy
DLB: Dementia with Lewy Bodies
IFA: Immuno Fluorescence Antibody assay
LBD: Lewy Body Disease
SLE: Systemic Lupus Erythematosus
CNS: Central Nervous System
IGs: Immunoglobulins
PS: Phosphatidylserine
